# Determining the preferred liquid reward in adult C57BL/6 mice

**DOI:** 10.1177/00236772221138628

**Published:** 2022-12-06

**Authors:** Amy L. Miller, Matthew C Leach

**Affiliations:** 1 School of Health and Life Sciences, 5462 Teesside University, UK; 2 School of Natural and Environmental Sciences, 5994 Newcastle University, UK

**Keywords:** Mouse, preference, reward, positive reinforcement, training

## Abstract

Using food rewards to motivate mice is commonplace in behavioural research. Using a reward which is highly desirable is of benefit, as it can reduce the need to food restrict animals and can encourage higher levels of consumption, allowing for changes in levels of consumption following an intervention to be easily determined. Here, we aimed to determine the preferred milk reward for adult male C57Bl/6 mice. Thirty male C57Bl/6 mice were provided with soya milk, chocolate milk, almond milk or water for a four-hour period in a cross-over design. When planning studies that involve the use of a palatable liquid reward, soya milk is the preferred option for adult male C57BL/6 mice. Providing a liquid reward of high value will give increased levels of consumption, with little or no food restriction required.

## Déterminer la récompense liquide préférée des souris C57BL/6 adultes Résumé

L’utilisation des récompenses alimentaires pour motiver les souris est un lieu commun dans la recherche comportementale. L’utilisation d’une récompense hautement souhaitable est un avantage car elle peut réduire le besoin de restriction alimentaire à imposer aux animaux et encourager des niveaux plus élevés de consommation, ce qui permet de déterminer facilement l’évolution de leur consommation à la suite d’une intervention. Nous cherchions ici à déterminer la récompense de lait préférée des souris C57Bl/6 mâles adultes. Trente souris mâles C57Bl/6 ont reçu du lait de soja, du lait au chocolat, du lait d’amande ou de l’eau pendant une période de 4 heures selon un plan croisé. Lors de la planification d’études impliquant l’utilisation d’une récompense liquide acceptable, le lait de soja est l’option préférée des souris C57BL/6 mâles adultes. Fournir une récompense liquide de haute valeur entraînera des niveaux accrus de consommation avec peu ou pas de restriction alimentaire nécessaire.

## Ermittlung bevorzugter flüssiger Belohnung bei adulten C57BL/6-Mäusen Abstract

In der Verhaltensforschung ist es üblich, Mäuse durch Belohnungen zu motivieren. Die Nutzung einer besonders erwünschten Belohnung ist von Vorteil, da sie die Notwendigkeit von Tieren mit Futterrestriktionen verringern und einen höheren Konsum fördern kann, so dass Veränderungen von Konsummengen nach einer Intervention leicht festgestellt werden können. In dieser Studie ging es um die Bestimmung der bevorzugten Belohnung in Milchform für adulte männliche C57Bl/6-Mäuse. Dreißig männliche C57Bl/6-Mäuse erhielten über einen Zeitraum von 4 Stunden in einem Cross-Over-Design entweder Sojamilch, Schokoladenmilch, Mandelmilch oder Wasser. Bei der Planung von Studien, die die Verwendung einer schmackhaften flüssigen Belohnung vorsehen, ist Sojamilch die bevorzugte Option für adulte männliche C57BL/6-Mäuse. Die Bereitstellung einer hochwertigen flüssigen Belohnung führt zu einer erhöhten Konsumrate, sodass wenig bis keine Futterbeschränkung erforderlich ist.

## Determinación de premios preferentes en formato líquido para roedores C57BL/6 adultos Resumen

El uso de premios de comida para motivar a los ratones es algo habitual en la investigación del comportamiento. Utilizar un premio que sea bastante deseable es beneficioso, ya que puede reducir la necesidad de restringir la alimentación de los animales y puede fomentar niveles más altos de consumo, permitiendo así determinar fácilmente los cambios en los niveles de consumo tras una intervención. Por tanto, nuestro objetivo es determinar un premio con la leche preferida de los ratones C57Bl/6 machos adultos. Treinta ratones C57Bl/6 macho recibieron leche de soja, leche de chocolate, leche de almendras o agua durante un periodo de 4 horas en un diseño cruzado. Cuando se planifican estudios que implican el uso de un premio apetitoso en formato líquido, la leche de soja es la opción preferida para los ratones C57BL/6 machos adultos. Proporcionar un premio líquido de alto valor hará que aumenten los niveles de consumo con poca o ninguna restricción alimentaria.

Mice are the most commonly used animals in scientific research, with more than a million used in regulated experimental procedures in the UK during 2019.^1^ Many behavioural studies require a mouse to perform a specific task, and motivation can be sought through the provision of a palatable food reward. Such studies include using operant conditioning to determine the willingness of a mouse to work to obtain a reward and compare the level of motivation to obtain the food with other types of reward, for example social interaction^2^ or nesting material.^3^ Neubert et al.^4^ developed an orofacial operant pain assay, whereby animals can make the choice between obtaining a liquid reward in the presence of a thermal nociceptive stimulus, or avoiding both the reward and the stimulus.^4^ Using this technique, the efficacy of various analgesics can be assessed in an orofacial pain model. A modified version of this has been successfully used in rats, whereby access to a liquid reward was available while the cheeks of the rat were in contact with a mechanical stimulus.^5^ Using this type of operant-based assay, the need for subjective assessment of the animals by a researcher is removed.

Determining the preferred type of liquid reward will have substantial benefits. For example, a very highly valued reward has the potential to result in the animals consuming it with little or no prior food restriction and would also increase the amount consumed to allow subtle changes in consumption to be more easily observed. Avoiding food restriction prior to data collection would result in reduced levels of stress^6^ and thus improved welfare and data quality.^7^ Additionally, if the reward is of significant value to the animal, the time taken to train animals to perform a task may reduce. The aim of our study was to determine the preferred liquid reward for adult male C57Bl/6 mice to use in behavioural assays.

Thirty male C57Bl/6 mice, specific pathogen free (Charles River Laboratories, Kent, UK), aged eight weeks on arrival were used in this study. Mice were housed in seven groups of four and one group of two in conventional open-top cages (38 cm × 50 cm × 18 cm) containing sawdust bedding (2HK Aspen wood chips; DBM, UK), paper nesting material, chew blocks, a plastic mouse house, two cardboard tubes and a Perspex handling tube. A seven-day acclimatisation period was given prior to the start of the study. The weights of the mice in each group at the start of the experiment are shown in Table 1. The animal room was maintained at 20 ± 2°C, 53% humidity, on a 12-hour/12-hour light/dark cycle (lights on at 02:00). Food (RM3 (E) PB)) and tap water were provided ad libitum at all times during the study. Experiments were approved by the Newcastle University Animal Welfare and Ethical Review Board. No regulated procedures which required a Home Office PPL were carried out as part of this data collection. The animals had not undergone any previous procedures.

**Table 1.** Cages were randomly allocated to one of four groups which determined the order in which the liquid rewards were presented to them across the four test days.

	Mean weight (g)	Test 1	Test 2	Test 3	Test 4
Group 1	26.7	Water	Almond milk	Soya milk	Chocolate milk
Group 2	25.2	Chocolate milk	Soya milk	Almond milk	Water
Group 3	25.3	Almond milk	Water	Chocolate milk	Soya milk
Group 4	26.0	Soya milk	Chocolate milk	Water	Almond milk

The test order and mean weight per mouse (in grams) for each group is shown.

Following acclimatisation, mice were exposed to three different types of liquid reward on four consecutive days in order to habituate them to the novel rewards. The liquid rewards selected were: chocolate milk (chocolate-flavoured 1% fat milk, Sainsbury’s, London, UK), soya milk (Alpro, Birmingham, UK) and almond milk (Alpro). Although sweetened condensed milk has been used in previous studies (e.g. Neubert et al.^4^), we opted not to use this in order to avoid the need to dilute the milk prior to use, and the nature of substance can result in water bottles becoming clogged. The various milks were presented in three separate ceramic ramekin dishes (7 cm × 7 cm × 4 cm), placed in the centre of the home cage between 11:00 and 15:00. This coincided with the end of the light phase/beginning of the dark phase when it would be expected that the mice would usually consume more food and water.^8^

The eight cages were then randomly allocated to one of four groups (two per group; Table 1) which determined the order in which they would be presented with the various milks during the test phase. Each test lasted for two consecutive days. On the first day, mice were weighed. Then, the allocated liquid reward was placed in a standard water bottle and presented to the mice in their home cage between 11:00 and 15:00. A second water bottle which always contained water was also present. Underneath both bottles, heavy ceramic dishes, which could not be tipped by the mice, were placed to catch any drips from the bottles. All water bottles were pretested to ensure that dripping was minimal. The presentation of the bottles was balanced so that four of the cages were given the test liquid on the left side of the cage, with water on the right. The other four cages had the bottles presented in the alternate configuration. On the second day, the test liquid and water bottles with drip trays were presented in exactly the same fashion, and the amount of liquid consumed from each bottle over the same four-hour period was determined at the cage level. The mice were then given a one-day break, and the two-day test period was then repeated, presenting each cage with a second test liquid as per the schedule in Table 1. This whole process was then repeated so that the volume of each test liquid consumed by each cage in a four-hour period was recorded. This cross-over design allowed mice to act as their own controls and account for any individual variation in liquid intake. Mice were weighed prior to each test to allow body weight to be factored into the analysis of liquid consumption.

Data were analysed using IBM SPSS Statistics for Windows v24 (IBM Corp., Armonk, NY). Data were normally distributed. So, a repeated-measures analysis of variance (ANOVA) was used to compare the consumption of liquid reward (g/kg bodyweight) at the cage level (*n* = 8 cages per group).

**Figure 1.**
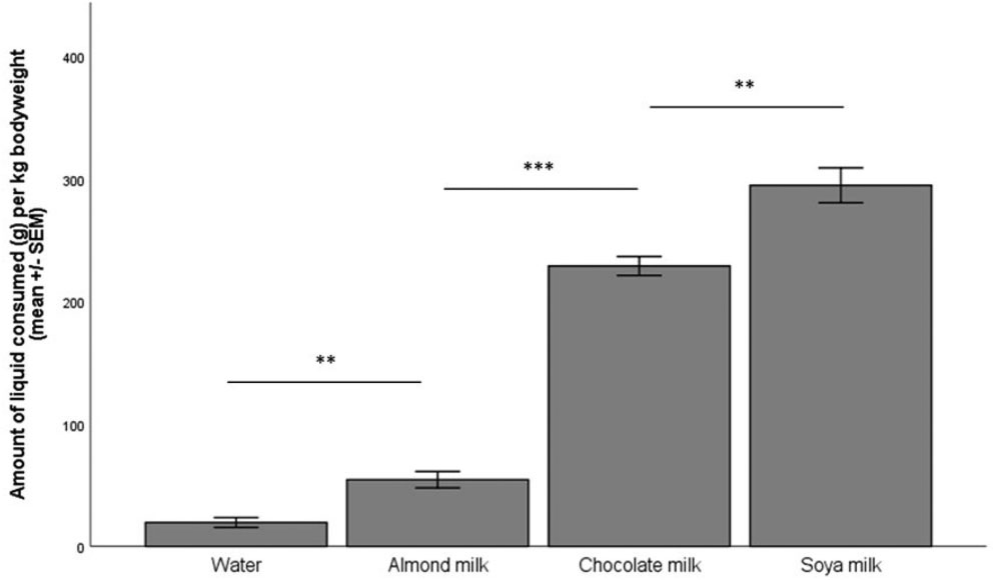
The amount (in grams) of the four different liquid rewards consumed per kilogram of body weight, *n* = 8 per group.

The repeated measures ANOVA showed a significant difference between the groups (*p* < 0.001). Soya milk was the preferred reward, and significantly more was consumed than chocolate milk (*p* = 0.002). Significantly more chocolate milk was consumed than almond milk (*p* < 0.001), and significantly more almond milk was consumed than water (*p* = 0.005) across the four-hour test period (Figure 1).

When carrying out behavioural trials that include the requirement for a palatable liquid reward, selecting one which is highly desirable is important. A reward of high value will improve motivation to perform a task and increase consumption, allowing changes in consumption following an intervention to be easily determined. Additionally, a reward of high value to the mice will reduce or negate the requirement to restrict food prior to experiments, reducing stress and thus improving welfare and the quality of the data collected. Mice are most active at dawn and dusk,^8^ and here we demonstrated that mice consumed significant amounts of soya milk in the hours surrounding the lights being turned off in the laboratory. Therefore, timings of experiments to coincide with the typical time at which mice are most active may be of benefit.
